# 7-Acetoxy­cochinchinone I^1^

**DOI:** 10.1107/S1600536809032292

**Published:** 2009-08-22

**Authors:** Suchada Chantrapromma, Nawong Boonnak, Hoong-Kun Fun

**Affiliations:** a Crystal Materials Research Unit, Department of Chemistry, Faculty of Science, Prince of Songkla University, Hat-Yai, Songkhla 90112, Thailand; b X-ray Crystallography Unit, School of Physics, Universiti Sains Malaysia, 11800 USM, Penang, Malaysia

## Abstract

The title compound {systematic name: 12-[(2*E*)-3,7-dimethyl-2,6-octa­dien­yl]-5,8-dihydr­oxy-2,2-dimethyl-2*H*,6*H*-pyrano[3,2-*b*]xanthen-6-one}, C_30_H_32_O_6_, has four fused rings (*A*/*B*/*C*/*D*) and the xanthone ring system (*A*/*B*/*C*) is essentially planar, with dihedral angles of 1.85 (13) and 2.47 (13)°, respectively, between rings *A* and *B*, and between rings *B* and *C*. The chromene ring *D* is in a sofa form. The geranyl side chain is axially attached to ring C with an (−)-synclinal conformation. The 3-methyl-2-butenyl terminal of the geranyl side chain is disordered with the site-occupancy ratio of 0.513 (5):0.487 (5). The acet­oxy group is attached axially to ring *A* with an (+)-synclinal conformation. An intra­molecular O—H⋯O hydrogen bond involving the carbonyl and hydroxyl groups generates an *S*(6) ring motif. In the crystal, weak C—H⋯O and C—H⋯π inter­actions, and π–π inter­actions with centroid–centroid distances of 3.6562 (16) and 3.6565 (16) Å are observed.

## Related literature

For hydrogen-bond motifs, see: Bernstein *et al.* (1995 ▶). For bond-length data, see: Allen *et al.* (1987 ▶). For ring conformations, see: Cremer & Pople (1975 ▶). For the bioactivity of xanthones, see, for examples: Boonnak *et al.* (2006 ▶, 2007 ▶, 2009 ▶); Molinar-Toribio *et al.* (2006 ▶); Vo (1997 ▶). For related structures, see, for example: Boonnak *et al.* (2009 ▶); Kosela *et al.* (1999 ▶). For the stability of the temperature controller used in the data collection, see: Cosier & Glazer, (1986 ▶).
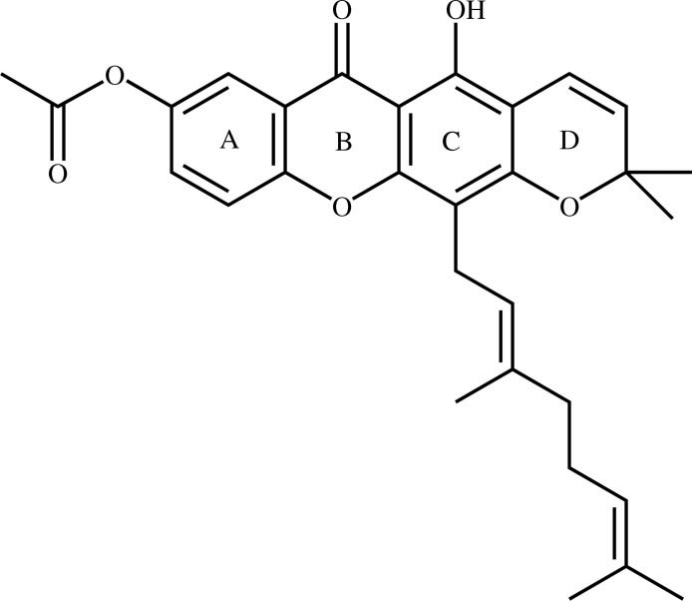


## Experimental

### 

#### Crystal data

C_30_H_32_O_6_
*M* *_r_* = 488.56
Triclinic, 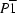

*a* = 6.1047 (1) Å
*b* = 8.6019 (1) Å
*c* = 25.4475 (4) Å
α = 97.705 (2)°
β = 91.888 (1)°
γ = 106.581 (1)°
*V* = 1265.60 (3) Å^3^
*Z* = 2
Mo *K*α radiation
μ = 0.09 mm^−1^
*T* = 100 K
0.37 × 0.14 × 0.07 mm

#### Data collection

Bruker APEXII CCD area-detector diffractometer
Absorption correction: multi-scan (**SADABS**; Bruker, 2005 ▶) *T*_min_ = 0.968, *T*_max_ = 0.994
20232 measured reflections
5745 independent reflections
4481 reflections with *I* > 2σ(*I*)
*R*_int_ = 0.056

#### Refinement

*R*[*F* ^2^ > 2σ(*F* ^2^)] = 0.095
*wR*(*F* ^2^) = 0.169
*S* = 1.22
5745 reflections
370 parameters
H-atom parameters constrained
Δρ_max_ = 0.26 e Å^−3^
Δρ_min_ = −0.22 e Å^−3^

### 

Data collection: *APEX2* (Bruker, 2005 ▶); cell refinement: *SAINT* (Bruker, 2005 ▶); data reduction: *SAINT*; program(s) used to solve structure: *SHELXTL* (Sheldrick, 2008 ▶); program(s) used to refine structure: *SHELXTL*; molecular graphics: *SHELXTL*; software used to prepare material for publication: *SHELXTL* and *PLATON* (Spek, 2009 ▶).

## Supplementary Material

Crystal structure: contains datablocks global, I. DOI: 10.1107/S1600536809032292/is2444sup1.cif

Structure factors: contains datablocks I. DOI: 10.1107/S1600536809032292/is2444Isup2.hkl

Additional supplementary materials:  crystallographic information; 3D view; checkCIF report

## Figures and Tables

**Table 1:** Hydrogen-bond geometry (Å, °)

*D*—H⋯*A*	*D*—H	H⋯*A*	*D*⋯*A*	*D*—H⋯*A*
O5—H1*O*5⋯O4	0.89	1.73	2.576 (3)	157
C5—H5*A*⋯O5^i^	0.93	2.58	3.275 (3)	132
C20—H20*A*⋯O4^ii^	0.96	2.47	3.285 (4)	143
C17—H17*A*⋯*Cg*3^iii^	0.97	2.74	3.694 (3)	171

Symmetry codes: (i) 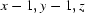; (ii) 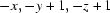; (iii) 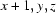. *Cg*3 is the centroid of the C1–C4/C11/C10 ring.

**Fig. 1.**
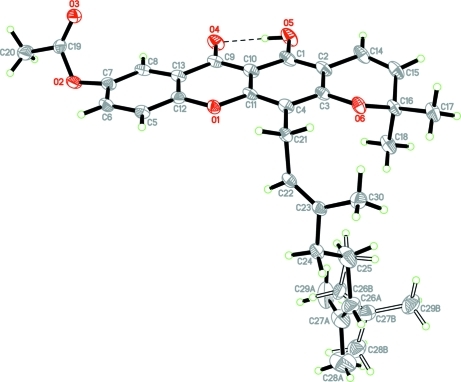
The structure of (I), showing 50% probability displacement ellipsoids and the atom-numbering scheme. Open bonds show the minor component. Hydrogen bonds were drawn as dash lines.

**Fig. 2.**
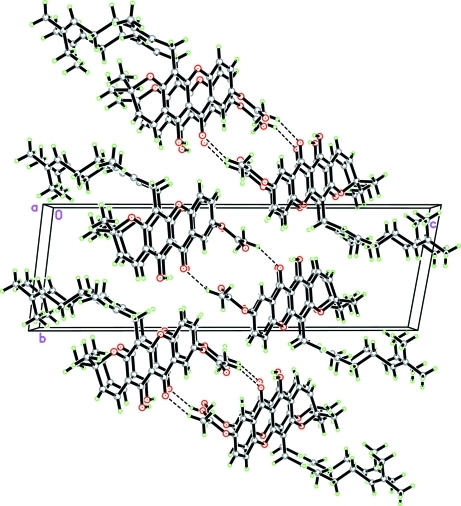
The crystal packing of the major component of the title compound, viewed along the a axis, showing the arrangement of the dimers into molecular sheets. Hydrogen bonds are shown as dashed lines.

## supplementary crystallographic information

### Comment

*Cratoxylum cochinchinense* belongs to the family Guttiferae, which is distributed in several parts of Thailand. This plant is a well known tropical tree and is commonly known in Thailand as "Tui Kliang". The bark, roots, and leaves of this plant have been used in folk medicine to treat fever, coughs, diarrhea, itches, ulcers, and abdominal complaints (Vo, 1997). Previous investigations revealed the major components from the *Cratoxylum* genus as xanthones and anthraquinones (Boonnak *et al.*, 2006, 2007, 2009). Xanthones have been reported to exhibit biological activities such as antibacterial and cytotoxic (Boonnak *et al.*, 2006, 2007, 2009) as well as antiprotozoal activities (Molinar-Toribio *et al.*, 2006). From our previous work it was found that cochinchinone I, the isolated xanthone from the resin of *Cratoxylum cochinchinense*, was active against *Pseudomonas aeruginosa* (Boonnak *et al.*, 2009). The title compound (I) is an acetylated product of cochinchinone I, which was modified in order to compare their antibacterial and cytotoxic activities. Herein we report the crystal structure of (I).

The title molecule (Fig. 1) has a four-fused rings (*A*/*B*/*C*/*D*), the xanthone skeleton is essentially planar, the dihedral angles between ring *A*/*B* and *B*/*C* are 1.85 (13) and 2.47 (13)°, respectively. The chromene ring *D* is in a sofa form with puckering parameter Q = 0.400 (3) Å, θ = 64.0 (4)° and φ = 314.5 (5)° (Cremer & Pople, 1975), the puckered C16 atom having the maximum deviation of -0.269 (3) Å. The two methyl groups are axially and bisectionally attached to chromine ring at atom C16 with torsion angles C14/C15/C16/C18 of 80.2 (3)° and C14/C15/C16/C17 of -155.0 (3)°, respectively. The geranyl side chain is axially attached to ring *C* at C4 with C3—C4—C21—C22 = -84.1 (3)°, indicating an (-)-*syn*-clinal conformation (Fig. 1). Moreover the 3-methyl-2-butenyl terminal of this geranyl side chain is disordered with the refined site-occupancy ratio of 0.513 (5)/0.487 (5). The acetoxy moiety is axially attached to ring *A* at C7 with C8—C7—O2—C19 = 60.2 (4)°, indicating an (+)-*syn*-clinal conformation and the dihedral angle between the mean plane through the acetoxy group [C19/C20/O2/O3] and ring *A* is 59.05 (14)°. O—H···O intramolecular hydrogen bond involving the carbonyl and hydroxyl moieties generates an S(6) ring motif (Bernstein *et al.*, 1995). The bond lengths in (I) show normal values (Allen *et al.*, 1987) and are comparable to the related structures; cochinchinone I (Boonnak *et al.*, 2009) and dulxanthone E (Kosela *et al.*, 1999).

In the crystal packing (Fig. 2), the symmetric weak C20—H20A···O4 interactions (Table 1) linked the molecules into dimers and generate *R*_2_^2^(18) motifs (Bernstein *et al.*, 1995). These dimers are arranged into molecular sheets parallel to the *bc* plane and these sheets are stacked along the *a* axis arising from π–π interactions with the *Cg*_1_···*Cg*_2_ distances of 3.6562 (16) Å (symmetry code: -1 + *x*, *y*, *z*) and 3.6565 (16) Å (symmetry code: 1 + *x*, *y*, *z*); *Cg*_1_ and *Cg*_2_ are the centroids of C1–C4/C10/C11 and C5–C8/C12/C13 rings, respectively. The crystal is also stabilized by a C—H···π interaction (Table 1).

### Experimental

The resin of *C. cochinchinense* (87.75 g) was extracted with CH_2_Cl_2_ (2 × 2.0 *L*, for a week) at room temperature and was evaporated under reduced pressure to afford a deep green crude CH_2_Cl_2_ extract (47.04 g), which was subjected to QCC (Quick Column Chromatography) on silica gel (Merck 60 F254) using hexane as a first eluent and then increasing the polarity with acetone to give 16 fractions (FR1—FR16). Fractions FR10 and FR11 were separated by QCC eluting with a gradient of acetone-hexane to give seven fractions (FR10A—FR10G). Subfraction FR10B was further purified by CC on silica gel C-18 and eluted with CH_3_OH to give cochinchinone I which was further converted to its derivative form by acetylation with Ac_2_O in pyridine to give the title compound, which was recrytalized out from CHCl_3_/CH_3_OH (9:1, *v*/*v*) to afford the yellow single crystals of the title compound after several days (m.p. 389–391 K).

### Refinement

Hydroxy H atom was located from the difference map and isotropically refined. The remaining H atoms were placed in calculated positions with d(C—H) = 0.93 Å for aromatic, 0.97 for CH_2_ and 0.96 Å for CH_3_ atoms. The *U*_iso_ values were constrained to be 1.5*U*_eq_ of the carrier atom for hydroxy and methyl H atoms and 1.2*U*_eq_ for the remaining H atoms. A rotating group model was used for the methyl groups. The highest residual electron density peak is located at 0.72 Å from C10 and the deepest hole is located at 0.45 Å from H14A.

### Crystal data

**Table:**

C_30_H_32_O_6_	*Z* = 2
*M**_r_* = 488.56	*F*(000) = 520
Triclinic, *P*1	*D*_x_ = 1.282 Mg m^−^^3^
Hall symbol: -P 1	Melting point = 389–391 K
*a* = 6.1047 (1) Å	Mo *K*α radiation, λ = 0.71073 Å
*b* = 8.6019 (1) Å	Cell parameters from 5745 reflections
*c* = 25.4475 (4) Å	θ = 2.4–27.5°
α = 97.705 (2)°	µ = 0.09 mm^−^^1^
β = 91.888 (1)°	*T* = 100 K
γ = 106.581 (1)°	Needle, yellow
*V* = 1265.60 (3) Å^3^	0.37 × 0.14 × 0.07 mm

### Data collection

**Table:**

Bruker APEXII CCD area-detector diffractometer	5745 independent reflections
Radiation source: sealed tube	4481 reflections with *I* > 2σ(*I*)
graphite	*R*_int_ = 0.056
φ and ω scans	θ_max_ = 27.5°, θ_min_ = 2.4°
Absorption correction: multi-scan (*SADABS*; Bruker, 2005)	*h* = −7→7
*T*_min_ = 0.968, *T*_max_ = 0.994	*k* = −11→11
20232 measured reflections	*l* = −33→33

### Refinement

**Table:**

Refinement on *F*^2^	Primary atom site location: structure-invariant direct methods
Least-squares matrix: full	Secondary atom site location: difference Fourier map
*R*[*F*^2^ > 2σ(*F*^2^)] = 0.095	Hydrogen site location: inferred from neighbouring sites
*wR*(*F*^2^) = 0.169	H-atom parameters constrained
*S* = 1.22	*w* = 1/[σ^2^(*F*_o_^2^) + (0.0391*P*)^2^ + 1.0353*P*] where *P* = (*F*_o_^2^ + 2*F*_c_^2^)/3
5745 reflections	(Δ/σ)_max_ < 0.001
370 parameters	Δρ_max_ = 0.26 e Å^−^^3^
0 restraints	Δρ_min_ = −0.22 e Å^−^^3^

### Special details

**Table:**

Experimental. The crystal was placed in the cold stream of an Oxford Cryosystems Cobra open-flow nitrogen cryostat (Cosier & Glazer, 1986) operating at 100.0 (1) K.
Geometry. All e.s.d.'s (except the e.s.d. in the dihedral angle between two l.s. planes) are estimated using the full covariance matrix. The cell e.s.d.'s are taken into account individually in the estimation of e.s.d.'s in distances, angles and torsion angles; correlations between e.s.d.'s in cell parameters are only used when they are defined by crystal symmetry. An approximate (isotropic) treatment of cell e.s.d.'s is used for estimating e.s.d.'s involving l.s. planes.
Refinement. Refinement of *F*^2^ against ALL reflections. The weighted *R*-factor *wR* and goodness of fit *S* are based on *F*^2^, conventional *R*-factors *R* are based on *F*, with *F* set to zero for negative *F*^2^. The threshold expression of *F*^2^ > σ(*F*^2^) is used only for calculating *R*-factors(gt) *etc*. and is not relevant to the choice of reflections for refinement. *R*-factors based on *F*^2^ are statistically about twice as large as those based on *F*, and *R*- factors based on ALL data will be even larger.

### Fractional atomic coordinates and isotropic or equivalent isotropic displacement parameters (Å^2^)

**Table:**

	*x*	*y*	*z*	*U*_iso_*/*U*_eq_	Occ. (<1)
O1	0.4903 (3)	0.0153 (2)	0.34364 (7)	0.0215 (4)	
O2	−0.2013 (3)	0.1650 (2)	0.45392 (8)	0.0274 (5)	
O3	0.0140 (3)	0.3410 (2)	0.52310 (8)	0.0290 (5)	
O4	0.5260 (3)	0.5024 (2)	0.37753 (8)	0.0303 (5)	
O5	0.8916 (3)	0.5734 (2)	0.32694 (9)	0.0338 (5)	
H1O5	0.7623	0.5744	0.3421	0.051*	
O6	1.1288 (3)	0.1277 (2)	0.24297 (8)	0.0249 (4)	
C1	0.8620 (5)	0.4101 (3)	0.31609 (12)	0.0275 (7)	
C2	1.0173 (5)	0.3570 (3)	0.28511 (12)	0.0261 (6)	
C3	0.9824 (4)	0.1868 (3)	0.27332 (11)	0.0223 (6)	
C4	0.8103 (5)	0.0702 (3)	0.29359 (11)	0.0225 (6)	
C5	0.1645 (5)	−0.0618 (3)	0.39063 (11)	0.0231 (6)	
H5A	0.1686	−0.1697	0.3833	0.028*	
C6	−0.0072 (5)	−0.0238 (3)	0.41912 (11)	0.0246 (6)	
H6A	−0.1205	−0.1062	0.4311	0.030*	
C7	−0.0089 (5)	0.1388 (3)	0.42974 (11)	0.0242 (6)	
C8	0.1559 (5)	0.2631 (3)	0.41349 (11)	0.0240 (6)	
H8A	0.1523	0.3710	0.4214	0.029*	
C9	0.5139 (5)	0.3543 (3)	0.36676 (11)	0.0244 (6)	
C10	0.6792 (5)	0.2985 (3)	0.33615 (11)	0.0243 (6)	
C11	0.6618 (4)	0.1303 (3)	0.32475 (11)	0.0209 (6)	
C12	0.3303 (5)	0.0636 (3)	0.37321 (10)	0.0220 (6)	
C13	0.3306 (5)	0.2261 (3)	0.38476 (11)	0.0235 (6)	
C14	1.2182 (5)	0.4675 (3)	0.26653 (13)	0.0318 (7)	
H14A	1.2611	0.5795	0.2789	0.038*	
C15	1.3398 (5)	0.4041 (4)	0.23125 (13)	0.0334 (7)	
H15A	1.4824	0.4677	0.2235	0.040*	
C16	1.2444 (5)	0.2300 (3)	0.20441 (11)	0.0243 (6)	
C17	1.4310 (5)	0.1556 (4)	0.18603 (12)	0.0308 (7)	
H17A	1.5389	0.1660	0.2155	0.046*	
H17B	1.5084	0.2121	0.1588	0.046*	
H17C	1.3640	0.0417	0.1721	0.046*	
C18	1.0709 (5)	0.2163 (4)	0.15850 (13)	0.0346 (7)	
H18A	0.9483	0.2564	0.1715	0.052*	
H18B	1.0103	0.1035	0.1426	0.052*	
H18C	1.1444	0.2802	0.1324	0.052*	
C19	−0.1699 (5)	0.2723 (3)	0.50082 (11)	0.0242 (6)	
C20	−0.3962 (5)	0.2855 (4)	0.51718 (13)	0.0369 (8)	
H20A	−0.3840	0.3261	0.5545	0.055*	
H20B	−0.5075	0.1794	0.5101	0.055*	
H20C	−0.4434	0.3597	0.4975	0.055*	
C21	0.7812 (5)	−0.1116 (3)	0.27952 (11)	0.0203 (6)	
H21A	0.9307	−0.1293	0.2771	0.024*	
H21B	0.7063	−0.1685	0.3074	0.024*	
C22	0.6412 (5)	−0.1810 (3)	0.22756 (11)	0.0210 (6)	
H22A	0.4926	−0.1726	0.2263	0.025*	
C23	0.7016 (5)	−0.2524 (3)	0.18336 (11)	0.0225 (6)	
C24	0.5354 (5)	−0.3151 (4)	0.13489 (11)	0.0292 (7)	
H24A	0.3826	−0.3189	0.1452	0.035*	
H24B	0.5344	−0.4264	0.1215	0.035*	
C25	0.5913 (6)	−0.2108 (5)	0.09019 (14)	0.0499 (10)	
H25A	0.7531	−0.1799	0.0861	0.060*	0.513 (6)
H25B	0.5456	−0.1130	0.0986	0.060*	0.513 (6)
H25C	0.7262	−0.2265	0.0748	0.060*	0.487 (6)
H25D	0.6274	−0.0978	0.1060	0.060*	0.487 (6)
C26A	0.4508 (14)	−0.3190 (11)	0.0352 (3)	0.0395 (18)	0.513 (6)
H26A	0.4791	−0.4172	0.0221	0.047*	0.513 (6)
C27A	0.2990 (14)	−0.2709 (9)	0.0089 (3)	0.0411 (18)	0.513 (6)
C28A	0.1669 (19)	−0.3770 (13)	−0.0404 (4)	0.069 (3)	0.513 (6)
H28A	0.2035	−0.4791	−0.0455	0.103*	0.513 (6)
H28B	0.2073	−0.3217	−0.0706	0.103*	0.513 (6)
H28C	0.0056	−0.3978	−0.0366	0.103*	0.513 (6)
C29A	0.2355 (14)	−0.1167 (10)	0.0241 (3)	0.060 (2)	0.513 (6)
H29A	0.3303	−0.0533	0.0548	0.090*	0.513 (6)
H29B	0.0775	−0.1434	0.0320	0.090*	0.513 (6)
H29C	0.2577	−0.0541	−0.0049	0.090*	0.513 (6)
C26B	0.4121 (13)	−0.2419 (10)	0.0485 (3)	0.0317 (17)	0.487 (6)
H26B	0.2618	−0.2663	0.0582	0.038*	0.487 (6)
C27B	0.4451 (14)	−0.2384 (8)	−0.0025 (3)	0.0322 (16)	0.487 (6)
C28B	0.2448 (16)	−0.2883 (12)	−0.0447 (3)	0.045 (2)	0.487 (6)
H28D	0.1039	−0.3268	−0.0283	0.067*	0.487 (6)
H28E	0.2627	−0.3742	−0.0709	0.067*	0.487 (6)
H28F	0.2416	−0.1953	−0.0615	0.067*	0.487 (6)
C29B	0.6724 (13)	−0.1817 (10)	−0.0245 (3)	0.046 (2)	0.487 (6)
H29D	0.7908	−0.1484	0.0040	0.069*	0.487 (6)
H29E	0.6766	−0.0906	−0.0429	0.069*	0.487 (6)
H29F	0.6966	−0.2697	−0.0488	0.069*	0.487 (6)
C30	0.9318 (5)	−0.2815 (4)	0.17768 (12)	0.0326 (7)	
H30A	1.0354	−0.2197	0.2074	0.049*	
H30B	0.9913	−0.2472	0.1453	0.049*	
H30C	0.9158	−0.3962	0.1768	0.049*	

### Atomic displacement parameters (Å^2^)

**Table:**

	*U*^11^	*U*^22^	*U*^33^	*U*^12^	*U*^13^	*U*^23^
O1	0.0212 (10)	0.0155 (9)	0.0258 (10)	0.0021 (7)	0.0038 (8)	0.0026 (8)
O2	0.0187 (10)	0.0284 (11)	0.0305 (11)	0.0043 (8)	−0.0016 (8)	−0.0051 (9)
O3	0.0274 (11)	0.0314 (11)	0.0239 (11)	0.0014 (9)	−0.0005 (9)	0.0060 (9)
O4	0.0288 (11)	0.0160 (10)	0.0433 (13)	0.0043 (8)	0.0008 (9)	0.0007 (9)
O5	0.0256 (11)	0.0141 (10)	0.0594 (15)	0.0019 (8)	0.0052 (10)	0.0053 (9)
O6	0.0197 (10)	0.0205 (10)	0.0343 (12)	0.0025 (8)	0.0039 (8)	0.0101 (8)
C1	0.0234 (15)	0.0145 (13)	0.0417 (18)	0.0007 (11)	−0.0038 (13)	0.0068 (12)
C2	0.0206 (14)	0.0203 (14)	0.0350 (17)	0.0010 (11)	−0.0049 (12)	0.0081 (12)
C3	0.0162 (13)	0.0228 (14)	0.0279 (15)	0.0060 (11)	−0.0035 (11)	0.0041 (12)
C4	0.0219 (14)	0.0173 (13)	0.0282 (15)	0.0051 (11)	−0.0023 (12)	0.0055 (11)
C5	0.0223 (14)	0.0197 (13)	0.0261 (15)	0.0057 (11)	−0.0014 (12)	0.0016 (11)
C6	0.0218 (14)	0.0201 (14)	0.0282 (16)	0.0005 (11)	−0.0008 (12)	0.0034 (12)
C7	0.0178 (14)	0.0264 (14)	0.0267 (15)	0.0070 (11)	−0.0023 (11)	−0.0021 (12)
C8	0.0222 (15)	0.0199 (14)	0.0274 (15)	0.0055 (11)	−0.0071 (12)	−0.0024 (11)
C9	0.0236 (15)	0.0192 (14)	0.0279 (16)	0.0042 (11)	−0.0055 (12)	0.0010 (11)
C10	0.0236 (15)	0.0190 (13)	0.0293 (16)	0.0048 (11)	−0.0039 (12)	0.0052 (12)
C11	0.0181 (14)	0.0154 (13)	0.0258 (15)	−0.0006 (10)	−0.0044 (11)	0.0044 (11)
C12	0.0214 (14)	0.0232 (14)	0.0196 (14)	0.0046 (11)	−0.0030 (11)	0.0024 (11)
C13	0.0215 (14)	0.0209 (14)	0.0250 (15)	0.0036 (11)	−0.0048 (11)	0.0005 (11)
C14	0.0215 (15)	0.0186 (14)	0.053 (2)	0.0004 (12)	0.0022 (14)	0.0097 (13)
C15	0.0215 (16)	0.0272 (16)	0.047 (2)	−0.0033 (12)	0.0021 (14)	0.0131 (14)
C16	0.0160 (14)	0.0255 (14)	0.0314 (16)	0.0026 (11)	0.0001 (12)	0.0129 (12)
C17	0.0211 (15)	0.0347 (16)	0.0377 (18)	0.0049 (12)	0.0038 (13)	0.0157 (14)
C18	0.0221 (16)	0.0402 (18)	0.0419 (19)	0.0040 (13)	−0.0002 (14)	0.0191 (15)
C19	0.0303 (16)	0.0207 (14)	0.0205 (15)	0.0035 (12)	0.0020 (12)	0.0081 (11)
C20	0.0298 (17)	0.0441 (19)	0.0340 (18)	0.0118 (15)	0.0021 (14)	−0.0065 (15)
C21	0.0198 (14)	0.0143 (12)	0.0263 (15)	0.0027 (10)	0.0021 (11)	0.0060 (11)
C22	0.0191 (14)	0.0151 (12)	0.0280 (15)	0.0013 (10)	0.0000 (11)	0.0083 (11)
C23	0.0210 (14)	0.0177 (13)	0.0258 (15)	−0.0005 (11)	0.0010 (11)	0.0061 (11)
C24	0.0228 (15)	0.0317 (16)	0.0287 (16)	0.0005 (12)	0.0027 (12)	0.0049 (13)
C25	0.036 (2)	0.068 (3)	0.041 (2)	−0.0003 (18)	−0.0023 (16)	0.0271 (19)
C26A	0.050 (5)	0.042 (5)	0.023 (4)	0.010 (4)	0.004 (3)	0.002 (3)
C27A	0.037 (4)	0.049 (4)	0.034 (4)	0.002 (3)	−0.005 (3)	0.019 (3)
C28A	0.074 (7)	0.080 (8)	0.038 (5)	−0.001 (6)	−0.022 (5)	0.020 (5)
C29A	0.048 (5)	0.080 (6)	0.067 (6)	0.030 (4)	0.023 (4)	0.033 (5)
C26B	0.032 (4)	0.040 (4)	0.024 (4)	0.013 (3)	0.009 (3)	0.003 (3)
C27B	0.032 (4)	0.033 (4)	0.032 (4)	0.013 (3)	−0.001 (3)	0.001 (3)
C28B	0.046 (5)	0.054 (6)	0.035 (5)	0.017 (4)	0.001 (4)	0.004 (4)
C29B	0.051 (5)	0.064 (5)	0.026 (4)	0.019 (4)	0.006 (3)	0.007 (3)
C30	0.0285 (17)	0.0382 (17)	0.0303 (17)	0.0109 (14)	0.0020 (13)	−0.0001 (13)

### Geometric parameters (Å, °)

**Table:**

O1—C11	1.369 (3)	C20—H20A	0.9600
O1—C12	1.375 (3)	C20—H20B	0.9600
O2—C19	1.379 (3)	C20—H20C	0.9600
O2—C7	1.404 (3)	C21—C22	1.502 (4)
O3—C19	1.192 (3)	C21—H21A	0.9700
O4—C9	1.247 (3)	C21—H21B	0.9700
O5—C1	1.352 (3)	C22—C23	1.323 (4)
O5—H1O5	0.8912	C22—H22A	0.9300
O6—C3	1.361 (3)	C23—C30	1.505 (4)
O6—C16	1.469 (3)	C23—C24	1.506 (4)
C1—C2	1.389 (4)	C24—C25	1.530 (4)
C1—C10	1.409 (4)	C24—H24A	0.9700
C2—C3	1.406 (4)	C24—H24B	0.9700
C2—C14	1.457 (4)	C25—C26B	1.440 (8)
C3—C4	1.396 (4)	C25—C26A	1.636 (8)
C4—C11	1.387 (4)	C25—H25A	0.9601
C4—C21	1.514 (3)	C25—H25B	0.9600
C5—C6	1.383 (4)	C25—H25C	0.9600
C5—C12	1.385 (4)	C25—H25D	0.9600
C5—H5A	0.9300	C26A—C27A	1.315 (11)
C6—C7	1.392 (4)	C26A—H26A	0.9300
C6—H6A	0.9300	C27A—C29A	1.492 (11)
C7—C8	1.363 (4)	C27A—C28A	1.505 (12)
C8—C13	1.398 (4)	C28A—H28A	0.9600
C8—H8A	0.9300	C28A—H28B	0.9600
C9—C10	1.444 (4)	C28A—H28C	0.9600
C9—C13	1.464 (4)	C29A—H29A	0.9600
C10—C11	1.409 (4)	C29A—H29B	0.9600
C12—C13	1.389 (4)	C29A—H29C	0.9600
C14—C15	1.341 (4)	C26B—C27B	1.324 (10)
C14—H14A	0.9300	C26B—H26B	0.9300
C15—C16	1.500 (4)	C27B—C29B	1.488 (10)
C15—H15A	0.9300	C27B—C28B	1.524 (11)
C16—C17	1.516 (4)	C28B—H28D	0.9600
C16—C18	1.520 (4)	C28B—H28E	0.9600
C17—H17A	0.9600	C28B—H28F	0.9600
C17—H17B	0.9600	C29B—H29D	0.9600
C17—H17C	0.9600	C29B—H29E	0.9600
C18—H18A	0.9600	C29B—H29F	0.9600
C18—H18B	0.9600	C30—H30A	0.9600
C18—H18C	0.9600	C30—H30B	0.9600
C19—C20	1.487 (4)	C30—H30C	0.9600
			
C11—O1—C12	119.8 (2)	C19—C20—H20B	109.5
C19—O2—C7	119.2 (2)	H20A—C20—H20B	109.5
C1—O5—H1O5	100.7	C19—C20—H20C	109.5
C3—O6—C16	116.5 (2)	H20A—C20—H20C	109.5
O5—C1—C2	118.1 (2)	H20B—C20—H20C	109.5
O5—C1—C10	120.4 (3)	C22—C21—C4	111.2 (2)
C2—C1—C10	121.5 (2)	C22—C21—H21A	109.4
C1—C2—C3	117.5 (3)	C4—C21—H21A	109.4
C1—C2—C14	123.7 (3)	C22—C21—H21B	109.4
C3—C2—C14	118.8 (3)	C4—C21—H21B	109.4
O6—C3—C4	116.0 (2)	H21A—C21—H21B	108.0
O6—C3—C2	120.1 (2)	C23—C22—C21	128.5 (3)
C4—C3—C2	123.8 (3)	C23—C22—H22A	115.7
C11—C4—C3	116.1 (2)	C21—C22—H22A	115.7
C11—C4—C21	122.4 (2)	C22—C23—C30	123.9 (3)
C3—C4—C21	121.4 (2)	C22—C23—C24	120.7 (3)
C6—C5—C12	118.9 (3)	C30—C23—C24	115.3 (2)
C6—C5—H5A	120.5	C23—C24—C25	113.7 (2)
C12—C5—H5A	120.5	C23—C24—H24A	108.8
C5—C6—C7	119.4 (3)	C25—C24—H24A	108.8
C5—C6—H6A	120.3	C23—C24—H24B	108.8
C7—C6—H6A	120.3	C25—C24—H24B	108.8
C8—C7—C6	122.0 (3)	H24A—C24—H24B	107.7
C8—C7—O2	122.0 (3)	C26B—C25—C24	115.7 (4)
C6—C7—O2	115.8 (2)	C24—C25—C26A	108.3 (4)
C7—C8—C13	119.1 (3)	C26B—C25—H25A	126.3
C7—C8—H8A	120.4	C24—C25—H25A	110.2
C13—C8—H8A	120.4	C26A—C25—H25A	110.2
O4—C9—C10	122.4 (3)	C26B—C25—H25B	80.8
O4—C9—C13	121.8 (3)	C24—C25—H25B	109.7
C10—C9—C13	115.9 (2)	C26A—C25—H25B	109.9
C1—C10—C11	117.7 (3)	H25A—C25—H25B	108.6
C1—C10—C9	121.2 (2)	C26B—C25—H25C	108.9
C11—C10—C9	121.2 (3)	C24—C25—H25C	108.7
O1—C11—C4	115.9 (2)	C26A—C25—H25C	86.1
O1—C11—C10	120.8 (2)	H25B—C25—H25C	130.4
C4—C11—C10	123.3 (3)	C26B—C25—H25D	108.2
O1—C12—C5	115.4 (2)	C24—C25—H25D	107.5
O1—C12—C13	123.1 (2)	C26A—C25—H25D	134.7
C5—C12—C13	121.5 (3)	H25A—C25—H25D	81.8
C12—C13—C8	119.1 (3)	H25C—C25—H25D	107.5
C12—C13—C9	119.3 (3)	C27A—C26A—C25	121.8 (8)
C8—C13—C9	121.7 (2)	C27A—C26A—H26A	119.1
C15—C14—C2	118.6 (3)	C25—C26A—H26A	119.1
C15—C14—H14A	120.7	C26A—C27A—C29A	125.8 (8)
C2—C14—H14A	120.7	C26A—C27A—C28A	120.2 (9)
C14—C15—C16	119.6 (3)	C29A—C27A—C28A	114.0 (8)
C14—C15—H15A	120.2	C27B—C26B—C25	124.9 (7)
C16—C15—H15A	120.2	C27B—C26B—H26B	117.5
O6—C16—C15	109.3 (2)	C25—C26B—H26B	117.5
O6—C16—C17	104.6 (2)	C26B—C27B—C29B	125.1 (7)
C15—C16—C17	112.2 (2)	C26B—C27B—C28B	121.5 (7)
O6—C16—C18	108.3 (2)	C29B—C27B—C28B	113.4 (6)
C15—C16—C18	111.3 (2)	C27B—C28B—H28D	109.5
C17—C16—C18	110.8 (3)	C27B—C28B—H28E	109.5
C16—C17—H17A	109.5	H28D—C28B—H28E	109.5
C16—C17—H17B	109.5	C27B—C28B—H28F	109.5
H17A—C17—H17B	109.5	H28D—C28B—H28F	109.5
C16—C17—H17C	109.5	H28E—C28B—H28F	109.5
H17A—C17—H17C	109.5	C27B—C29B—H29D	109.5
H17B—C17—H17C	109.5	C27B—C29B—H29E	109.5
C16—C18—H18A	109.5	H29D—C29B—H29E	109.5
C16—C18—H18B	109.5	C27B—C29B—H29F	109.5
H18A—C18—H18B	109.5	H29D—C29B—H29F	109.5
C16—C18—H18C	109.5	H29E—C29B—H29F	109.5
H18A—C18—H18C	109.5	C23—C30—H30A	109.5
H18B—C18—H18C	109.5	C23—C30—H30B	109.5
O3—C19—O2	123.1 (3)	H30A—C30—H30B	109.5
O3—C19—C20	127.6 (3)	C23—C30—H30C	109.5
O2—C19—C20	109.3 (2)	H30A—C30—H30C	109.5
C19—C20—H20A	109.5	H30B—C30—H30C	109.5
			
O5—C1—C2—C3	−178.9 (2)	C6—C5—C12—O1	−177.8 (2)
C10—C1—C2—C3	1.5 (4)	C6—C5—C12—C13	1.5 (4)
O5—C1—C2—C14	4.1 (4)	O1—C12—C13—C8	177.6 (2)
C10—C1—C2—C14	−175.5 (3)	C5—C12—C13—C8	−1.7 (4)
C16—O6—C3—C4	155.6 (2)	O1—C12—C13—C9	−2.6 (4)
C16—O6—C3—C2	−28.0 (3)	C5—C12—C13—C9	178.1 (3)
C1—C2—C3—O6	179.8 (2)	C7—C8—C13—C12	0.5 (4)
C14—C2—C3—O6	−3.0 (4)	C7—C8—C13—C9	−179.3 (3)
C1—C2—C3—C4	−4.1 (4)	O4—C9—C13—C12	−177.7 (3)
C14—C2—C3—C4	173.1 (3)	C10—C9—C13—C12	2.0 (4)
O6—C3—C4—C11	179.7 (2)	O4—C9—C13—C8	2.1 (4)
C2—C3—C4—C11	3.4 (4)	C10—C9—C13—C8	−178.2 (3)
O6—C3—C4—C21	−3.7 (4)	C1—C2—C14—C15	−171.4 (3)
C2—C3—C4—C21	−179.9 (3)	C3—C2—C14—C15	11.6 (4)
C12—C5—C6—C7	−0.2 (4)	C2—C14—C15—C16	11.3 (4)
C5—C6—C7—C8	−0.9 (4)	C3—O6—C16—C15	47.3 (3)
C5—C6—C7—O2	173.6 (2)	C3—O6—C16—C17	167.6 (2)
C19—O2—C7—C8	−60.2 (4)	C3—O6—C16—C18	−74.1 (3)
C19—O2—C7—C6	125.3 (3)	C14—C15—C16—O6	−39.3 (4)
C6—C7—C8—C13	0.8 (4)	C14—C15—C16—C17	−155.0 (3)
O2—C7—C8—C13	−173.4 (2)	C14—C15—C16—C18	80.2 (3)
O5—C1—C10—C11	−178.1 (3)	C7—O2—C19—O3	−2.5 (4)
C2—C1—C10—C11	1.5 (4)	C7—O2—C19—C20	177.3 (2)
O5—C1—C10—C9	2.6 (4)	C11—C4—C21—C22	92.4 (3)
C2—C1—C10—C9	−177.8 (3)	C3—C4—C21—C22	−84.1 (3)
O4—C9—C10—C1	−1.0 (4)	C4—C21—C22—C23	119.2 (3)
C13—C9—C10—C1	179.3 (3)	C21—C22—C23—C30	1.6 (4)
O4—C9—C10—C11	179.7 (3)	C21—C22—C23—C24	179.9 (2)
C13—C9—C10—C11	0.0 (4)	C22—C23—C24—C25	107.2 (3)
C12—O1—C11—C4	−177.6 (2)	C30—C23—C24—C25	−74.4 (3)
C12—O1—C11—C10	1.3 (4)	C23—C24—C25—C26B	−166.2 (5)
C3—C4—C11—O1	178.7 (2)	C23—C24—C25—C26A	163.1 (4)
C21—C4—C11—O1	2.1 (4)	C26B—C25—C26A—C27A	9.2 (7)
C3—C4—C11—C10	−0.2 (4)	C24—C25—C26A—C27A	119.4 (7)
C21—C4—C11—C10	−176.8 (2)	C25—C26A—C27A—C29A	0.6 (12)
C1—C10—C11—O1	179.0 (2)	C25—C26A—C27A—C28A	−177.7 (7)
C9—C10—C11—O1	−1.7 (4)	C24—C25—C26B—C27B	−143.2 (7)
C1—C10—C11—C4	−2.2 (4)	C26A—C25—C26B—C27B	−61.7 (9)
C9—C10—C11—C4	177.1 (3)	C25—C26B—C27B—C29B	−8.0 (12)
C11—O1—C12—C5	−179.8 (2)	C25—C26B—C27B—C28B	174.2 (7)
C11—O1—C12—C13	0.9 (4)		

### Hydrogen-bond geometry (Å, °)

**Table:**

*D*—H···*A*	*D*—H	H···*A*	*D*···*A*	*D*—H···*A*
O5—H1O5···O4	0.89	1.73	2.576 (3)	157
C5—H5A···O5^i^	0.93	2.58	3.275 (3)	132
C20—H20A···O4^ii^	0.96	2.47	3.285 (4)	143
C17—H17A···Cg3^iii^	0.97	2.74	3.694 (3)	171

Symmetry codes:  (i) *x*−1, *y*−1, *z*; (ii) −*x*, −*y*+1, −*z*+1; (iii) *x*+1, *y*, *z*.
